# A rare case of eosinophilic cystitis involving the inside and outside of the urinary bladder associated with an infected urachal cyst

**DOI:** 10.1186/s12894-021-00885-6

**Published:** 2021-08-30

**Authors:** Hyun Bin Shin, Hyun Sik Park, Joo Heon Kim, Jinsung Park

**Affiliations:** 1 Department of Urology, Eulji University Hospital, Eulji University School of Medicine, Daejeon, Korea; 2 Department of Pathology, Eulji University Hospital, Eulji University School of Medicine,, Daejeon, Korea; 3 Department of Urology, Uijeongbu Eulji Medical Center, Eulji University School of Medicine, 712, Dongil-ro, Uijeongbu-si, Gyeonggi-do 11759 Republic of Korea

**Keywords:** Eosinophilic cystitis, Urachal cyst, Partial cystectomy

## Abstract

**Background:**

Eosinophilic cystitis is a rare inflammatory disease of the bladder characterized by eosinophilic infiltration of the bladder wall. Most Eosinophilic cystitis cases present with mucosal lesions of the urinary bladder. We present a very rare case of large mass-forming eosinophilic cystitis, involving the inside and outside of the bladder associated with an infected urachal cyst.

**Case presentation:**

A 59-year-old man presented with gross hematuria, fever, dysuria, and suprapubic pain. Computed tomography showed a heterogeneously enhancing mass that measured 7.6 cm × 4 cm located on the anterosuperior portion of the bladder with an internal fluid collection. Cystoscopy revealed a raspberry-like mass lesion on the bladder dome. Transurethral resection of the bladder was initially performed. The mass lesion protruding from inside the bladder was removed, and pus-like fluid was drained. The pathologic diagnosis was eosinophilic cystitis. Follow-up computed tomography showed a remnant mass outside the bladder and urachal cyst. To eliminate the remnant lesion, robot-assisted partial cystectomy was performed. The patient showed no evidence of recurrent disease on follow-up cystoscopy and computed tomography for up to 2 years.

**Conclusions:**

Clinicians should consider the possibility of eosinophilic cystitis in patients who present with hematuria, fever, and suprapubic pain and have both intravesical and extravesical masses.

## Background

Eosinophilic cystitis (EC) is a rare inflammatory disease of the bladder, characterized by eosinophilic infiltration of the bladder wall and associated with fibrosis with or without muscle necrosis [1]. Because of the small number of patients afflicted with EC, the etiology of EC is not well understood. But subsequent mast cell degranulation is thought to be due to antigenic stimulation that promotes IgE-mediated attraction of eosinophils throughout the bladder wall [2–5]. The most common symptoms include urinary frequency, hematuria, dysuria, urinary retention, and suprapubic pain.

Most EC cases present with mucosal lesions of the urinary bladder [6, 7], although several studies have reported mass-forming or malignancy-mimicking EC [1, 2, 6–9]. To diagnose EC, pathologic diagnosis of bladder mucosal lesions such as transmural eosinophil infiltration and absence of malignant cells by transurethral resection of the bladder (TURB) or cystoscopic biopsy, is essential [1, 2, 4, 6, 7]. To date, there is only one case report on EC associated with infected urachal remnants [8]; but the patient was a 4-year-old child diagnosed with EC at 7 months after surgical excision of the infected urachal cyst, with recurrent abscesses in the abdomen, pelvis, and at the operation site. In our case report, we present a rare case of large mass-forming EC, involving both the inside and outside of the urinary bladder associated with an infected urachal cyst.

## Case presentation

A 59-year-old man presented with gross hematuria, fever (37.9 °C), dysuria, and suprapubic pain at the Eulji University Hospital. The patient had no history of allergies or bladder injury, and had an unremarkable medical history. Laboratory tests showed elevation in c-reactive protein levels (17.31 mg/dL) but no leukocytosis (7.810 × 10^3^/μL), elevation of erythrocyte sedimentation rate (ESR, 6 mm/h), and peripheral eosinophilia (270 cells/μL). Urinalysis revealed hematuria, but no bacterial growth was found in urine culture.

**Fig. 1:**
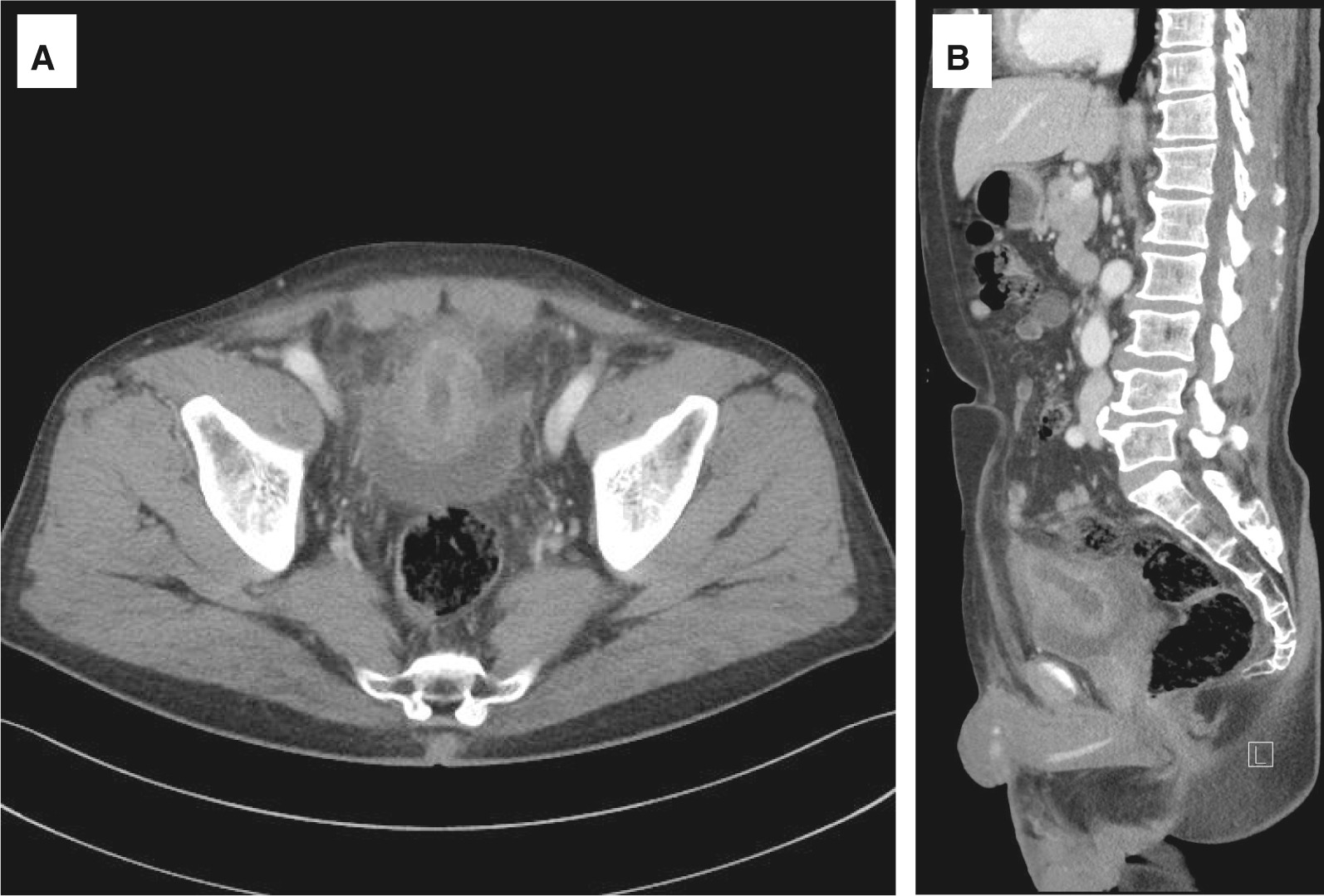
Computed tomography showing a heterogeneous enhancing mass on the anterosuperior portion of the bladder. **A** Axial view, **B** sagittal view

**Fig. 2:**
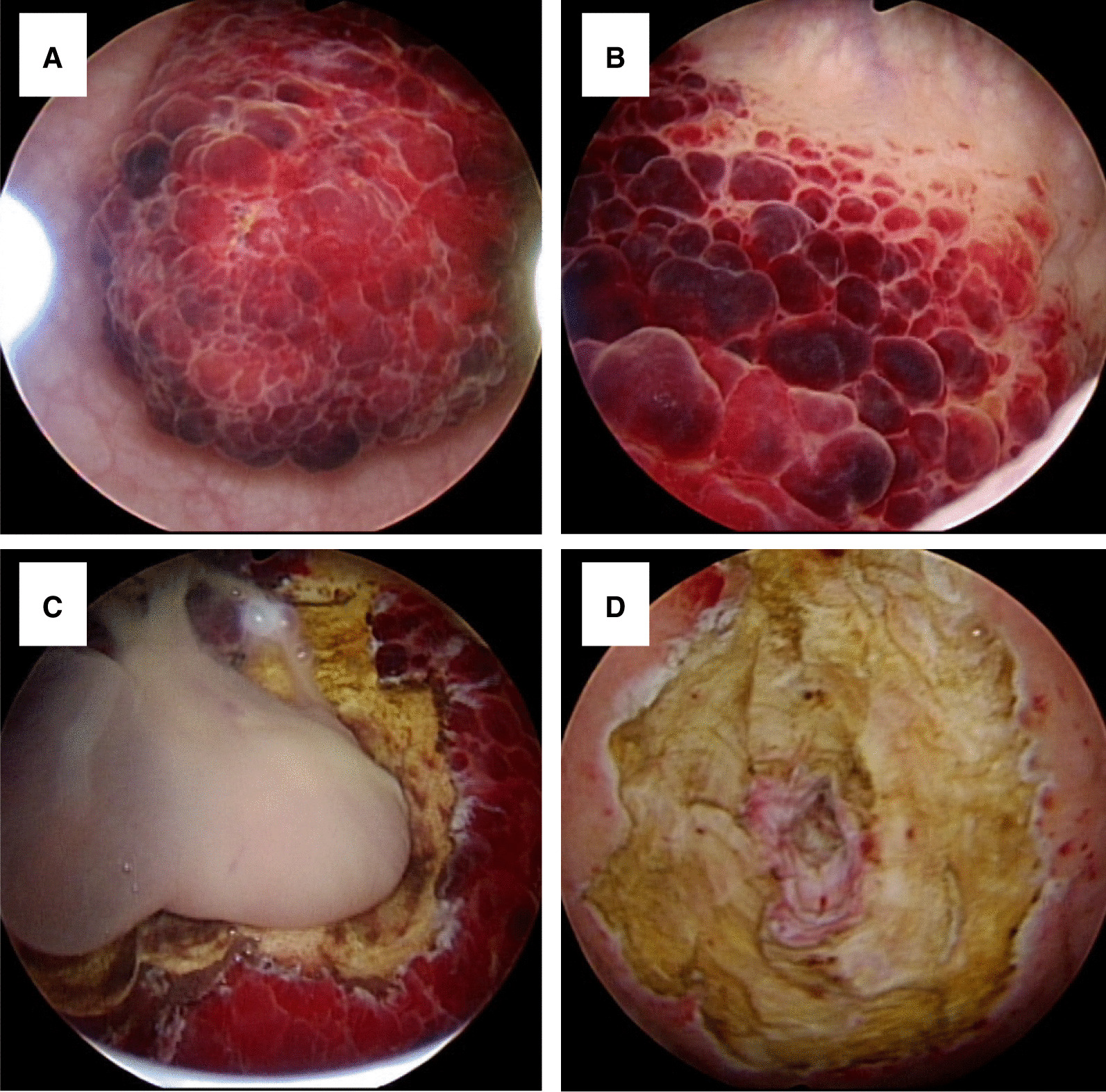
Cystoscopic findings of bladder lesion. **A**, **B** Raspberry-like lesion on bladder dome. **C** At transurethral resection of the bladder, pus-like fluid is visible after resection of the mass lesion. **D** Tumor bed after mass resection. After resection of the mass lesion inside the bladder, the lesion outside the bladder is observed in the center

Computed tomography (CT) showed a heterogeneously enhancing mass that measured 7.6 cm × 4 cm on the anterosuperior portion of the bladder (Fig. 1). The mass had a sphere-like shape and a thickened outer wall with an internal fluid collection. Initially, an infected urachal cyst was suspected because the patient had a fever, and empirical intravenous antibiotics (third-generation cephalosporin) were initiated. Cystoscopy showed a huge raspberry-like mass lesion on the bladder dome with a definite margin, which occupied more than half of the bladder (Fig. 2A, B). Because the possibility of a tumorous condition could not be fully excluded, percutaneous drainage was not performed. After infection control with intravenous antibiotics, TURB was performed for pathologic diagnosis. During resection, a pus-like fluid was found inside the mass (Fig. 2C), and most mass lesions inside the bladder were removed (Fig. 2D). Tissue and fluid culture were performed, but no bacterial growth was observed.

**Fig. 3:**
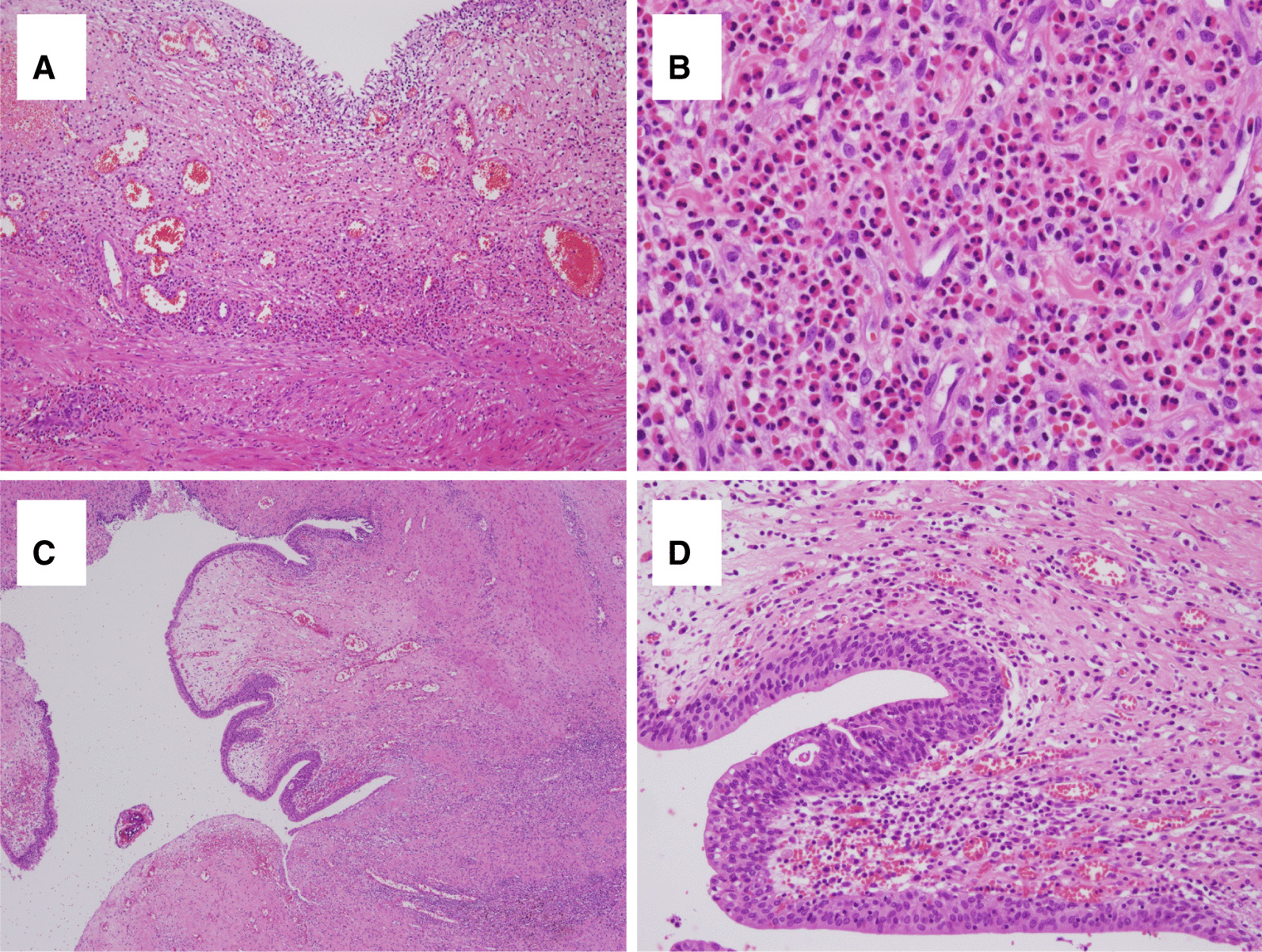
Histologic findings of partial cystectomy specimen. **A** Low magnification photomicrography showing subepithelial and perivascular inflammatory cell infiltrate in the bladder (H&E. ×100). **B** Histopathology showing many eosinophils in the bladder wall (H&E. ×400). **C** The infected urachal cyst lined by urothelium and surrounded by mixed inflammatory cell infiltrate. (H&E. ×40). **D** The infected urachal cyst, histopathology showing numerous lymphocytes and plasma cells (H&E. ×200)

Histopathological examination revealed numerous eosinophilic infiltrates in the bladder wall. These were diffusely infiltrated and gathered around vessels in the lamina propria and muscularis propria. Mild urothelial papillary hyperplasia was present. No viral inclusions were observed. Immunohistochemical staining showed patchy positive reaction on CK20 and CD 44 proteins. The tumor protein P53 showed score 1 (less than 5%) positivity. The pathologic diagnosis was EC (Fig. 3).

**Fig. 4:**
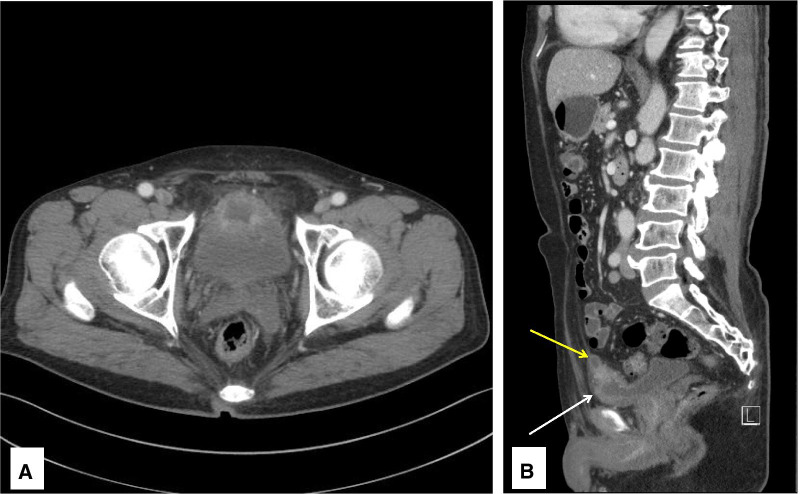
Follow-up computed tomography after transurethral resection shows heterogeneous enhancing mass, reduced in size, on the anterosuperior portion of the bladder. **A** Axial view of bladder mass. **B** Sagittal view of remnant bladder mass (white arrow) with an urachal cyst (yellow arrow)

**Fig. 5:**
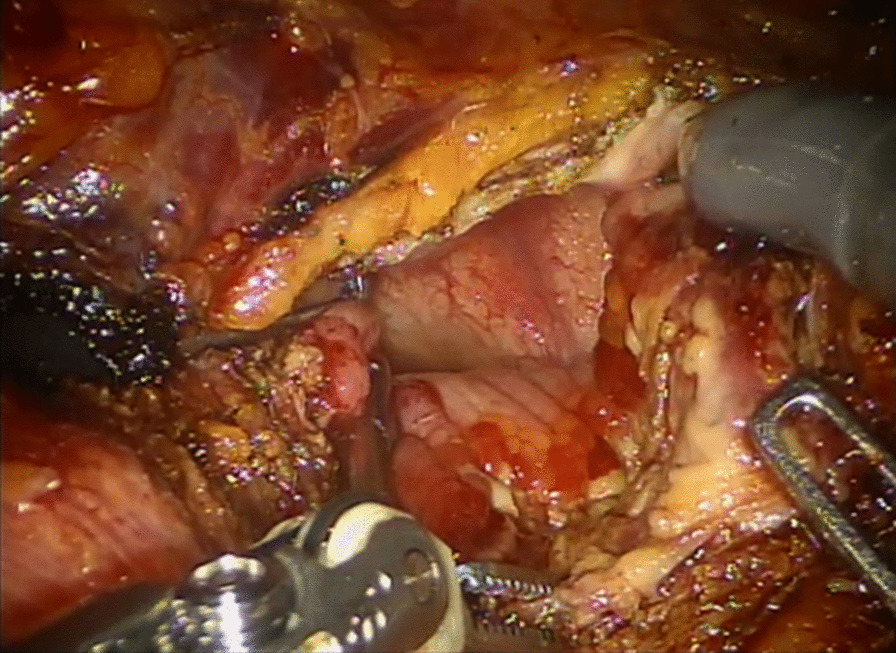
Intraoperative view of robot-assisted partial cystectomy. Remnant lesion of eosinophilic cystitis after transurethral resection of the bladder (TURB) is identified at the bladder dome, i.e., remnant ulcerative, and a raspberry-like lesion is still visible. The lesion is completely removed with the infected urachal cyst, and the bladder is closed with continuous running suture

At 2 weeks postoperatively, follow-up CT showed shrinkage of the mass lesion, but a remnant ulcerative lesion, which was thought to be the outer part of the mass lesion, was still noted at the bladder anterior and dome side (Fig. 4A). Another 1.4-cm cystic mass, thought to be the urachal cyst, was incidentally detected above the remnant mass (Fig. 4B). Thus, the mass that was resected with a resectoscope had EC, and the infected urachal cyst existed above the mass. To eliminate the remnant EC lesion and urachal cyst, robot-assisted partial cystectomy was performed (Fig. 5). The urachal cyst was removed with a whole layer of the bladder wall that was, associated with the EC lesion. Similar to that seen in TURB lesions, partial cystectomy specimens showed heavy eosinophilic infiltration on the whole layers of the bladder wall, mucosal erosion, and fibrosis in the subepithelial connective tissue. An infected urachal cyst is accompanied by numerous lymphocytes, plasma cells, and histiocytic infiltrates. The benign epithelium of the urachal cyst is made up of cuboidal-type urothelium.

After partial cystectomy, the patient recovered uneventfully and was discharged on postoperative day 7. The patient was treated with antihistamine (hydroxyzine hydrochloride) for 15 months and regularly followed up. There was no evidence of recurrence in the follow-up CT and cystoscopy for up to 23 months.

## Discussion and conclusion

To the best of our knowledge, this is the first report on EC that formed mass lesions involving both the inside and outside of the urinary bladder associated with infected urachal cysts. The majority of EC cases present with erythematous bladder mucosal lesions or bullous edematous lesions without a definite cause [4, 5, 7, 10, 11]. Although a few patients have been reported to present with bladder tumor-mimicking lesions [4, 5, 7, 10, 12], there is a case report of a pediatric patient who had a bladder tumor-mimicking lesion wherein the same was found after surgical excision of the infected urachal remnant [8]. In our case report, the patient presented with a sphere-like mass involving the inside and outside of the bladder as well as an infected urachal cyst. When the patient was initially presented, the bladder lesion was difficult to diagnose with EC because a huge berry-looking mass mimicking a bladder tumor was visible. To confirm the pathologic diagnosis and distinguish malignancy, TURB was performed.

Histopathological examination revealed heavy infiltration of eosinophils around vessels in the lamina propria and muscularis propria of the bladder mucosa. The diagnosis of EC can be made when many eosinophils are visible in bladder specimens [1, 2, 4, 6, 7]. There are no definite pathologic criteria for EC, but more than 25 eosinophils per high power field of view can be thought of as indicating EC, similar to eosinophilic esophagitis and gastritis [13]. In our case, heavy infiltration of eosinophils was visible around the mucosa and vessels in the lamina propria and muscularis propria, therefore diagnosis of EC could be made.

In laboratory findings, EC usually shows hematuria or proteinuria [14]. Furthermore, peripheral eosinophilia was observed in 42.5% to > 50% of the patients, and elevated ESR was observed in 7% of the patients [1, 7, 14]. In our case, the patient only showed hematopyuria without bacterial growth, and no elevation of ESR and peripheral eosinophilia was observed.

The etiology of EC is not well known because of the small number of cases. In most cases, allergies or bladder injuries are present [6]. Since EC is a result of antigen–antibody complexes [4, 5], avoiding antigens is also an effective method to treat EC. However, finding antigen is difficult in most cases, aside from using bladder instillation therapy. In our case, the patient had no allergies or history of bladder injury, but an infected urachal cyst was incidentally detected. Infected urachal cysts can act as allergens and attract eosinophils to the bladder wall. The infected urachal cyst may also have caused bladder injury due to the mass effect. Hypothetically, bladder inflammation induced by an infected urachal cyst could be the cause of EC. However, because the urachal cyst in our case was small (maximum diameter 1.7 cm) and the patient had no specific history (allergy, medical history, or bladder injury), it is not clear whether the small urachal cyst caused EC in this patient.

There is no definite curative treatment for EC, but medical treatments such as corticosteroids, antihistamines, and antibiotics can be considered as the primary treatment [3–5, 7, 14–16]. Corticosteroids can help control inflammation and symptoms, and antihistamines can help suppress the inflammatory reaction, which inhibits the formation of immune complexes. Antibiotics can be used when EC is associated with urinary tract infection. In our case, antibiotics were initially used because the mass was thought to be related to an infected cystic lesion, and antihistamines were used to prevent recurrence after partial cystectomy.

If corticosteroids or antihistamines could not resolve EC, surgical treatment is also an option [1, 5, 7, 16–18]. Surgical treatment not only has a high treatment success rate, but also a low recurrence rate [4, 7]. In our case, the reason for choosing partial cystectomy was that it required removal of the remnant urachal cyst. On CT, both urachal cysts and EC lesions were visible after TURB, and partial cystectomy was a good option to remove both lesions in one surgery.

Although the prognosis of EC is not well known, recurrences are common even after treatment. In a pooled analysis involving 135 patients, among 59 patients who received medical treatment, 10 patients (17.0%) showed recurrence of EC after medical treatment [7], whereas only 1 patient (2.6%) showed recurrence after surgery among 38 patients who were treated with surgery. However, in generalized lesions, medical treatment should be accompanied by surgical treatment [4]. Therefore, to prevent recurrence, our patient was treated with antihistamines (hydroxyzine hydrochloride) for 15 months. After 15 months, CT and cystoscopy showed no recurrence, and we decided to discontinue treatment with hydroxyzine hydrochloride and closely monitor the patient with CT. The patient was followed-up for 23 months without evidence of recurrence.

In conclusion, clinicians should consider the possibility of EC in patients presenting with hematuria, fever, and suprapubic pain with a bladder mass. Pathologic diagnosis can be made using cystoscopic biopsy or TURB, and this is essential to distinguish malignancy. In many cases, medical treatment shows successful outcomes, but partial cystectomy can be safely performed in patients with large mass-forming EC associated with urachal cyst, without any major complications.

## Data Availability

All data generated or analysed during this study are included in this published article.

## Abbreviations

EC: Eosinophilic cystitis
TURB: Transurethral resection of the bladder tumor
CT: Computed tomography
